# The role of gut microbiota in the formation of IBD, a typical chronic intestinal inflammatory disease

**DOI:** 10.3389/fmicb.2025.1720709

**Published:** 2025-12-17

**Authors:** Jinglin Chen

**Affiliations:** Jiangyou People’s Hospital, Jiangyou, Sichuan, China

**Keywords:** gut microbiota, colitis, inflammatory bowel disease, genetic factors, environmental factors, immune regulation

## Abstract

The existing etiological studies of inflammatory bowel disease (IBD) mainly focus on genetics, environment, immunity and gut microbiota (GM). Interestingly, this review found that the first three causes are not separate, but closely related to GM. First, the genetic characteristics of GM are closely related to the pathogenesis of IBD, and multiple human genomic loci (such as LCT and ABO) have been identified to affect GM changes and increase the risk of IBD. Secondly, the composition of GM is directly related to the environment at birth. With the growth of human beings, environmental factors such as diet, antibiotics, and even psychosocial stress can lead to significant changes in the composition and function of the GM, thus affecting the intestinal inflammatory state of the host. In addition, GM disruption stimulates the body to accumulate a large number of inflammatory cells and activates the immune system to repair tissue while resisting infection, resulting in colitis. Therefore, this review takes genetic factors, environmental factors, and immune regulation as entry points to explore the important role of GM in the formation of colitis and the possible benefits of regulating GM composition, providing new strategies for the prevention and treatment.

## 1 Introduction

Inflammatory bowel disease (IBD) is a family of conditions characterized by chronic, relapsing inflammation of the gastrointestinal tract. The etiology and pathogenesis of IBD have not been completely clarified yet, and the existing etiological studies on IBD mainly focus on environmental, genetic, immune and the gut microbiota (GM) factors (Zuo and Ng, 2018). Among them, GM plays a significant role in the pathogenesis of IBD and is thought to have complex interactions with other factors.

Adverse alterations in the composition and function of GM are known as dysbiosis, which alters host–microbiota interaction and the host immune system. Dysbiosis of the GM, characterized by an altered ratio of pro-inflammatory to anti-inflammatory microorganisms, plays a central role in triggering and perpetuating intestinal damage (Wu N. et al., 2021). Because it is estimated that 70% of the human microbiota is located in the colon (Sekirov et al., 2010), and GM can be involved in the synthesis of inflammation (Moskal et al., 2016). For example, a relative increase in the abundance of the Proteobacteria was consistently found in the GM of IBD patients, especially in pro-inflammatory *Escherichia coli* (*E. coli*), an adherent-invasive *E. coli* that increases intestinal epithelial permeability and induces intestinal inflammation through direct adhesion to it (Nishida et al., 2021). However, the increase of probiotic *Bifidobacterium fragilis* can promote the secretion of short-chain fatty acids (SCFAs) to negatively regulate the NLRP3-mediated inflammatory signaling pathway, inhibit the secretion of pro-inflammatory mediators such as IL-18 and IL-1β, reduce the level of intestinal inflammation (Shao et al., 2021). It can be seen that the disorder of GM is closely related to the occurrence of IBD. Therefore, this review aims to clarify the influencing factors and changes of GM in the formation of chronic colitis, so as to promote the importance of GM in the prevention of colitis.

## 2 Influencing factors of GM in the pathogenesis of (chronic) colitis

### 2.1 Genetic factors

Chronic inflammatory bowel disease IBD is thought to be the result of a persistent inflammatory process against endogenous microorganisms in genetically susceptible individuals and has been shown to be an inherited disease that exhibits familial aggregation. First-degree relatives of infected individuals have a 4–8-fold increased risk of IBD; Children born to couples with concurrent IBD are at much higher risk; Families containing 2 or more members affected by IBD become more prevalent; And identical twins both have an increased probability of suffering from IBD compared to heterozygous twins (Gordon et al., 2022; Torres et al., 2022; Santos et al., 2018). Also twin, family and population studies have demonstrated the heritability of certain GM, so that the genetic characteristics of GM are inextricably linked to the development of IBD.

In recent years, a large number of studies have begun to analyze the influence of host genetics on the human GM, and Kurilshikov et al. (2021) identified 19 heritable microbiota and 31 motifs affecting the microbiota, among which the lactase gene (*LCT*) and the fucosyltransferase 2 gene (*FUT2*) have an important influence in shaping our gut ecosystem; Qin et al. (2022) identified three genetic loci closely associated with GM variation, namely the *LCT*, *ABO* and mediator complex subunit 13L gene (*MED13L*). Genome-wide association studies (GWAS) have provided evidence for a genetic contribution to microbiome composition, and an increasing number of loci have been identified to have an impact on GM alterations and increase the risk of colitis-related diseases. However, only two motifs (*LCT* and *ABO*) have been consistently replicated in at least three studies. Among them, genetic variants at or near the *LCT* locus were found to be associated with *Actinomyces*, *Bifidobacterium* and their related species. Genetic variants at or near the *ABO* locus were associated with the abundance of *E. coli*, *Bacteroides* and *Bifidobacterium* (Schmidt et al., 2020; Bonder et al., 2016). In addition, to explore the potential impact of genetic-GM associations on host health-related characteristics, researchers analyzed the potential causal relationship between GM and disease phenotypes by Mendelian randomization. Zhang et al. (2021) determined that Akkermansia (*p* = 0.05) and Dorea (*p* = 0.04) were causally associated with the development of IBD. Liu et al. (2022) determined that Coprococcus 2 (*p* = 0.022), Oxalobacter (*p* = 0.001). and Ruminococcaceae UCG014 (*p* = 0.005) were positively associated with the risk of developing IBD; Genus Enterorhabdus (*p* = 0.044) and *Eubacterium ventriosum* (*p* = 0.011) were negatively associated with the risk of developing IBD. Kurilshikov et al. (2021) found that the abundance of Bifidobacterium may have a protective effect against colitis. It can be seen that the genetic variation of host genes has a certain contribution to the composition and abundance of GM.

### 2.2 Environmental factors

First, studies on human subjects have shown that GM development begins immediately after birth and that GM composition is directly related to the environment encountered by infants. The composition of an infant’s microbiota is initially defined by the mode of delivery—infants born by cesarean section acquire a skin-based microbiota, whereas infants born vaginally acquire a vaginal microbiota (Maaser et al., 2017). Differences in early gut colonization can affect subsequent GM development, increasing the risk of specific diseases. The probability of hospitalization for gastrointestinal disorders, especially IBD, is higher in offspring delivered by cesarean section (*p* = 0.004) (Zamstein et al., 2022). A study assessing the microbiota of children 7 years after delivery showed that children born by cesarean section had a significantly lower abundance of *Clostridium* (*p* = 0.0055) (Salminen et al., 2004), which is involved in the metabolism of tryptophan and usually exerts a protective effect against IBD. At later stages of life, environmental factors such as diet (Wark et al., 2020), smoking, antibiotics, and even psychosocial stress can lead to significant changes in the composition and function of the GM (Shouval and Rufo, 2017). Modulating the GM through a diet rich in vegetables, legumes, grains, nuts, and fish, as well as through a higher intake of plant-based than animal-based foods, could prevent intestinal inflammation and thus inhibit the development of many chronic diseases. These dietary changes can reduce the abundance of *E. coli*, *Bacteroides fragilis* (*B. fragilis*), and *Parabacteroides* and increase the abundance of *Bifidobacterium* (Bolte et al., 2021). Similarly, smoking can decrease the diversity of beneficial gut microbes. In one study, an increased abundance of *Bacteroidetes* and a reduced abundance of *Firmicutes* were observed in the fecal microbiota of smokers versus never smokers (Papoutsopoulou et al., 2020; Lee et al., 2018). These alterations were similar to the GM changes seen in IBD patients (Gui et al., 2021). Antibiotics can also reduce microbiota diversity, for example, by reducing *Bifidobacterium* and butyrate-producing species, thus increasing the probability of IBD (Ramirez et al., 2020). With the discovery of the “brain–gut–microbiome” axis, researchers have found that psychological activity and emotional changes can also affect the composition of the GM and thus influence the inflammatory state of the host gut. Meanwhile, animal studies have demonstrated that a sterile environment prevents colitis in genetically susceptible mice, while the transfer of pro-inflammatory microbes from diseased mice to healthy mice induces inflammation (Glassner et al., 2020). For example, Ohkusa et al. (2003) isolated *Fusobacterium varium* from the colonic mucosa of patients with ulcerative colitis and found that the butyric acid in the supernatant of the bacterial culture could induce ulcerative colitis in experimental models. However, recent hygiene hypotheses for the development of immune diseases suggest that raising children in an extremely hygienic environment with reduced chances of parasitic infections may negatively impact immune system development, predisposing them to immune diseases such as IBD. This is because helminths may induce beneficial changes in the GM (significantly decreasing the abundance of *Bacteroides* and increasing the abundance of *Clostridium*) to reduce the intestinal inflammatory response (Ramanan et al., 2016). Thus, the environmental impact on the GM is two-sided but is a key factor in the development of chronic intestinal inflammatory diseases.

### 2.3 Immune regulation

There are two main levels of immune regulation in the intestine. First, intestinal epithelial cells, mucosa, and the host microbiota create a physical barrier that provides the first layer of protection for the symbiotic relationship between the GM and the host. Second, once the antigenic substances cross the first line of defense and enter the organism causing GM dysbiosis, specific immunity represented by T cells and B cells in the body can form an internal barrier against intestinal invaders, providing a second layer of protection (Schirmer et al., 2019). Intestinal inflammation is caused by dysfunction at the above two levels, causing the GM to enter a state of imbalance.

**Figure 1:**
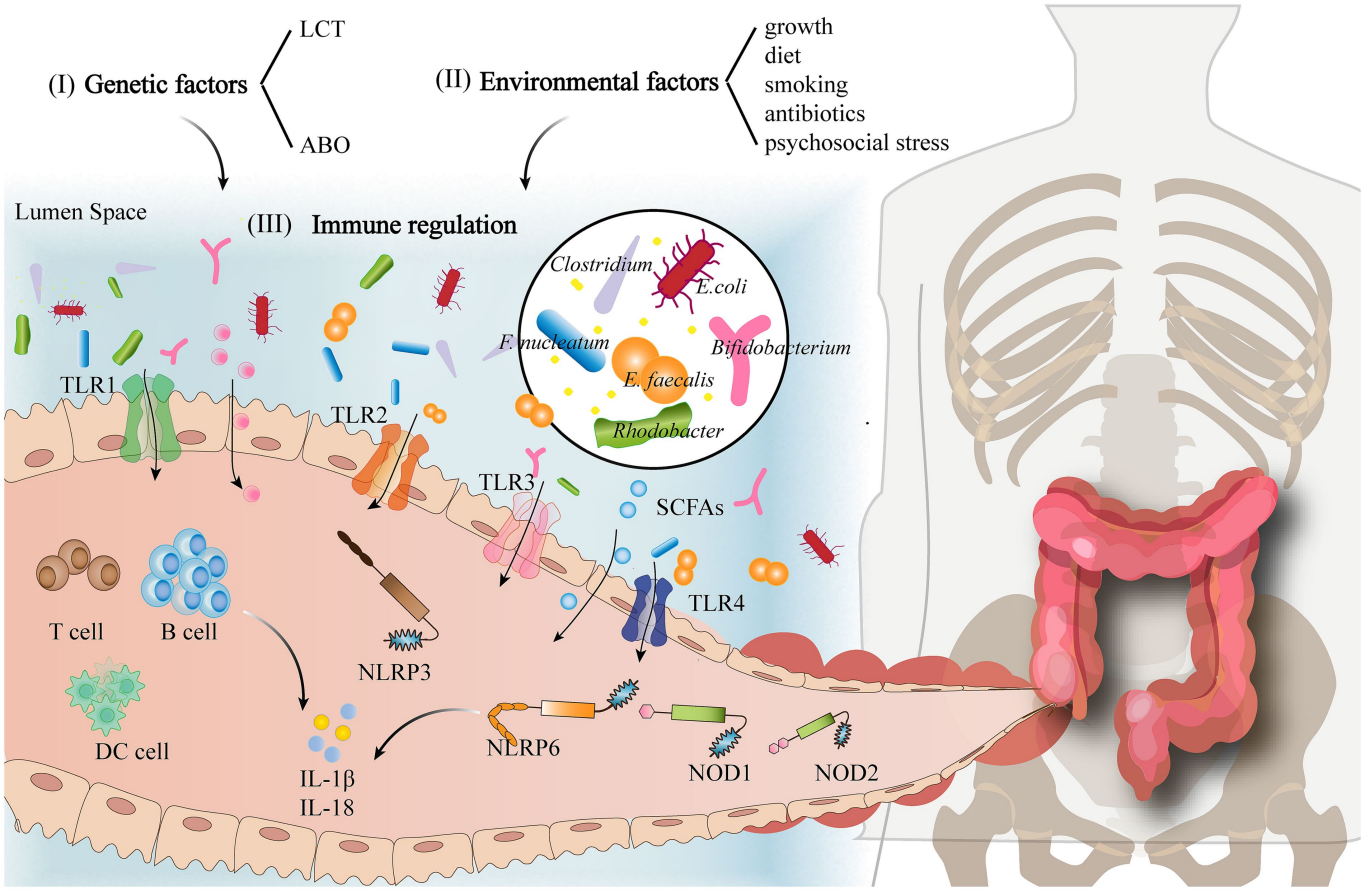
Influencing factors of GM in the pathogenesis of (chronic) colitis.

Epithelial damage is the initiating event in dextran sodium sulfate (DSS)-induced colitis model, and intestinal permeability is an important indicator of colitis severity (Wirtz et al., 2017). Epithelial cell defects are often observed in animals with chronic inflammatory intestinal diseases. Mouse models of chronic colitis infected with *Campylobacter coli* show mild or moderate histopathological changes within the colonic mucosa as well as the apoptosis of colonic epithelial cells. Notably, probiotics can enhance the intestinal barrier by modulating bacterial products such as peptides and SCFAs to increase the thickness of the colonic mucus layer. For example, Bian et al. (2020) treated DSS-induced mouse models of colitis with *Pediococcus pentosaceus* LI05. This significantly increased the abundance of *Anaerofilum*, *Ruminiclostridium*, *Faecalibacterium*, and *Akkermansia*, enabling GM regulation to maintain epithelial barrier function, reduce host inflammation, and increase SCFA production, contributing to the alleviation of DSS-induced colitis. However, studies have shown that if a pathogen invades the intestine, the GM immediately activates both nonspecific and specific immune mechanisms to counteract it. Non-specific immunity involves a physiological rejection of multiple antigens by the body, and it typically involves pattern recognition receptors (PRRs) that recognize potential pathogens and harmless antigens. Of these PRRs, Toll-like receptors (TLRs) and NOD-like receptors (NLRs) are important protein molecules involved in non-specific immunity and serve as the bridge between non-specific and specific immunity. TLRs are single transmembrane non-catalytic proteins that recognize molecules with conserved structures derived from microorganisms. When microorganisms cross the body’s physical barriers, such as the skin and mucous membranes, TLRs can recognize them and activate a cellular immune response. The expression of TLR2 and TLR4 is directly associated with the abundance of *Fusobacterium nucleatum* (*F. nucleatum*), *Enterococcus faecalis* (*E. faecalis*), *Streptococcus bovis* (*S. bovis*), and *Porphyromonas* (Rezasoltani et al., 2020). In addition, the TLR2 monoclonal antibody (TLR2mAb) and TLR4 monoclonal antibody (TLR4mAb) can inhibit the development of DSS-induced colitis and increase the abundance of *Lactobacillus* and *Bifidobacterium* (Dong et al., 2012). TLR3 and TLR5 expression is directly correlated with the number of *Bifidobacterium*, *Rhodobacter*, and *Lactobacillus*. Hence, the interaction between the GM and TLRs affects immune regulation and inflammation, thereby influencing homeostasis and immune responses *in vivo* (Rezasoltani et al., 2020). NLRs are a type of nonspecific immune receptors located in the intracellular environment and initiate inflammatory processes. NOD1, NOD2, NLRP6, and NLRP12 are members of the NLR family. NOD1 prevents the expansion of certain intestinal bacteria such as *Clostridium*, *Bacteroides*, *segmented filamentous bacteria* (SFB), and *Enterobacteriaceae*. NOD2 receptors control commensal microbiota and mediate the elimination of pathogenic bacteria from intestinal crypts, which minimizes the risk of intestinal inflammation. NLRP3-deficient mice exhibit significantly altered abundances of gut microbes such as *Enterobacteriaceae* and *Clostridium* (Hirota et al., 2011). NLRP6 receptor deficiency decreases IL-1β and IL-18 levels, thereby promoting intestinal dysbiosis and increasing the risk of colitis. In addition, commensal microbes can activate NLRP6 inflammatory vesicles, promoting mucus production in cuprocytes and antimicrobial peptides that maintain a healthy GM. Similarly, NLRP12 receptor deficiency promotes colonic inflammation, decreases GM diversity, and increases colon-derived bacteria (Chen et al., 2017; Elias-Oliveira et al., 2020; Guo et al., 2020). Specific immunity refers to a series of defensive functions that arise postnatally through contact with antigens. The adaptive immunity induced by a dysbiosis of the GM mainly occurs at sites such as intestine-associated lymphoid tissues. Macrophages, dendritic cells (DCs), T cells, and B cells in the lamina propria of the gut also play an important role in specific immunity. Dysregulated numbers of *Campylobacter coli* in mice enhance the adaptive immune cell response, resulting in increased numbers of macrophages and monocytes as well as regulatory T and B lymphocytes in the colonic mucosa and lamina propria, as well as enhanced systemic tumor necrosis factor-α (TNF-α) secretion (Heimesaat et al., 2020). It is evident that GM-mediated immune regulation plays an important role in the prevention of autoimmune and inflammatory diseases. On the one hand, the GM can influence the formation and maturation of intestinal mucosal immunity to prevent the invasion of exogenous pathogens. On the other hand, once pathogen invasion occurs, the GM immediately initiates non-specific and specific immune mechanisms to counteract it (see Figure 1).

## 3 GM in (chronic) colitis prevention strategy

Numerous studies have confirmed that an imbalance of the GM is a key factor in the progression of IBD. However, it is not clear which specific bacteria show significant alterations during the induction of enteritis. In recent studies, the most studied component of the GM is the bacterial microbiota. Notably, 98% of intestinal bacteria belong to four major bacterial phyla, namely, *Bacteroidetes*, *Firmicutes*, *Proteobacteria*, and *Actinobacteriota*. The altered diversity of intestinal microorganisms in IBD patients also involves these four phyla, including a decrease in the abundance of *Firmicutes*, especially changes in its subordinate genus *Lactobacillus* and an increase in the abundance of *Enterobacteriaceae* family in the phylum *Proteobacteria*. Some studies have also observed changes in *Bacteroides* and *Bifidobacterium* in the *Actinobacteriota* family. Given the above-mentioned relationships between the GM and environmental, and immunomodulatory factors, it appears that the genera *Bacteroides*, *Clostridium*, *Lactobacillus*, *Enterobacteriaceae*, and *Bifidobacterium* from the four major bacterial phyla play an important role in the regulation of (chronic) intestinal inflammation (Table 1).

**Table 1:** Changes and immune mechanisms of four major bacterial phyla in (chronic) intestinal inflammatory hosts.

Bacterial phyla	Bacterial genera	Immune mechanism	References
*Bacteroidetes*	*Bacteroides*↓ (decrease for genus)	Sphingolipids produced by *Bacteroides* can inhibit inflammation and are essential for maintaining intestinal homeostasis and symbiosis	Brown et al. (2019)
		*Bacteroides* can relieve colonic inflammation by mechanisms involved in the regulation of tryptophan metabolism and T cell subsets in inflammatory intestinal tissues	Li et al. (2021)
		*Bacteroides* can modulate the host immune response by changing the activity of the transcription factor NF-κB and inhibiting the activation of downstream inflammatory factors (such as IL-8, TNF-α, and IL-1β)	Delday et al. (2019)
		Extracellular vesicles produced by *Bacteroides* interact with TLR4 in a specific manner to influence host immunity	Gul et al. (2022)
		*Bacteroides* colonization in sterile mice can promote the maturation of the colon immune system, especially the highly induced Treg pathway	Hoffmann et al. (2016)
*Firmicutes*	*Clostridium*↑ (increase for genus)	The human *Clostridium* strain VE202 has an IL-10 independent protection mechanism, which corrects ecological imbalance by reducing the levels of *Enterobacteriaceae* and *Fusobacterium*	Oka et al. (2020)
		The co-culture of *Clostridium difficile* with human intestinal microvascular endothelial cells can inhibit the proliferation of intestinal mucosal cells and promote their apoptosis, reduce the expression of the AQP1 protein, and inhibit the permeability of intestinal cells	Hui et al. (2018)
		*Clostridium difficile* produces enterotoxins TcdA and TcdB, which bind to specific receptors on colonic epithelial cells and affect the integrity of the intestinal barrier by disrupting epithelial tight junctions, recruiting neutrophils and activating pro-inflammatory cytokines to promote inflammation	Boeriu et al. (2022)
	*Lactobacillus*↓ (decrease for genus)	*Lactobacillus* is an important element of breast milk, which has a positive effect on the composition of the intestinal flora and intestinal immune system and can alleviate IBD symptoms	Abdi et al. (2021)
		*Lactobacillus* can induce the antigen-specific proliferation of T cells *in vitro*	Weingarden et al. (2022)
		The synbiotic combination of prebiotic grape residue extract and probiotic *Lactobacillus* has anti-inflammatory properties and can downregulate the expression of most inflammatory genes, proteins, and related signal markers	Pistol et al. (2019)
		Mixed lactobacilli show better anti-inflammatory effects than single treatment, because they increase bacterial diversity, improve intestinal microflora composition, and enhance SCFA production	Shi et al. (2021)
		The probiotic combination of *Lactobacillus* and *Bifidobacterium* can not only improve the disease phenotype but also restore the composition and structure of the intestinal microbiota and reduce the symptoms of intestinal inflammation	Xu et al. (2022)
*Proteobacteria*	*Enterobacteriaceae*↑ (increase for genus)	*Enterobacteriaceae* contains molecular components that are located on the bacterial surface and directly enhance the inflammatory response. These molecules interact with receptors on immune cells to trigger inflammation	Scales et al. (2016)
		There are unmethylated immunostimulatory motifs in *Enterobacteriaceae* that induce immune responses by interacting with TLR9	Hemmi et al. (2000)
		Mice lacking TLR5 and IL-10 develop spontaneous infectious colitis associated with the abnormal expansion of *Enterobacteriaceae*	Carvalho et al. (2012) and Maharshak et al. (2013)
		*Enterobacter ludwigii* was found to induce the immune tolerance of DCs to Treg differentiation by upregulating retinoic acid (RA) and TGF-β via choline and α7nAChR, thereby protecting mice from DSS-induced colitis	Li et al. (2022)
		*E. coli* producing α-hemolysin (HlyA) damages intestinal barrier function through focal epithelial leakage, thereby enhancing antigen uptake and triggering intestinal inflammation in susceptible mouse models	Bücker et al. (2014)
		*E. coli* can induce the secretion of IL-8, TNF-α, and IL-1β, destroy the intestinal mucosa, and increase intestinal permeability and inflammation	Mirsepasi-Lauridsen et al. (2019)
*Actinobacteriota*	*Bifidobacterium*↓ (decrease for genus)	*Bifidobacterium* relieves the symptoms of IBD by enhancing antioxidant activity and regulating the production and accumulation of ROS to regulate oxidative stress	Yao et al. (2021)
		*Bifidobacterium* can reduce DSS-induced colitis by enhancing the intestinal barrier and producing anti-inflammatory effects through the AhR pathway	Cui et al. (2022)
		*Bifidobacterium* alleviates spontaneous and chemical-induced colitis by regulating cytokines or inducing specific immunomodulatory mechanisms (for example, *Bifidobacterium infantis* can accelerate the expression of Foxp3 in the intestine by activating the PD-1/PD-L1 signaling pathway. It can also promote the expression of IL-10 and TGF-β1 to reduce intestinal inflammation and has a therapeutic effect in IBD mouse models)	Kim et al. (2022) and Zhou et al. (2022)

Accordingly, potential strategies for the prevention and treatment of (chronic) inflammatory bowel diseases focus on modulating the composition and function of the aforementioned gut microbes. Currently, microbiome-dependent colitis prevention and treatment is based on dietary changes and modulation of the immune system. Dietary changes, such as dietary supplementation with probiotics, which can modify the composition of the GM, are the most common. Breast milk containing the probiotic *Lactobacillus* can alter the composition of the GM, and breastfeeding for more than 6 months reduces the risk of colitis. The oral administration of *Bacillus subtilis*-fermented milk can alleviate DSS-induced IBD by inhibiting the inflammatory response, promoting mucosal barrier reconstruction, and regulating the GM. In addition, the extracellular polysaccharide fraction of *Bacillus subtilis* can be used as a functional food to improve intestinal barrier function and reduce colonic inflammation. Therefore, fermented foods containing edible microorganisms have been explored as dietary supplements for the pretreatment of IBD. Further, synbiotic supplements that combine probiotics and prebiotics attenuate the side effects of commonly used dietary supplements, which demonstrates their importance in the pretreatment of colitis. However, the composition of the human GM is very complex, and only a limited number of bacterial strains can be artificially produced. More than 60% of gut microbes cannot be cultured at present. Thus, probiotic products can only be improved at the food level and cannot directly restore the balance of the GM. One solution is fecal microbiota transplantation (FMT) technology. In this process, the normal GM is isolated from the feces of healthy individuals and is transplanted into the intestine of patients with dysbiosis to enhance the survival of beneficial bacteria, improve intestinal immunity, and rebuild a normal intestinal microecological system that can prevent and alleviate colitis. The effectiveness and safety of FMT in treating *Clostridium difficile* infection (CDI) in IBD patients have been recognized (Ianiro et al., 2021). In addition, most patients with inflammatory intestinal diseases exhibit an impaired immune system. Hence, altering the abundance of probiotic and pathogenic bacteria in the GM can modulate the host immune system and promote both specific and non-specific immunity. For example, the expression of inflammatory factors and activation of signaling pathways to restore intestinal homeostasis and prevent or alleviate colonic inflammation can be achieved via the TLR-dependent recognition of intestinal bacteria.

Although it is challenging to construct a more accurate disease prediction model by combining genetic information and gut microbiota data, genetic factors are gradually becoming the core pillar to guide its personalized treatment in the future IBD management. For example, on the basis of understanding the genetic background of patients, we can use the “polygenic risk score” and “dysbacteriosis index” to comprehensively evaluate the incidence and severity of IBD in patients, and then adjust the environmental factors (especially diet and lifestyle) in advance for high-risk groups (such as individuals with a family history of IBD and obvious dysbacteriosis detected by GM test), and select the most suitable flora intervention target according to the immune characteristics of patients, so as to realize the accurate remodeling of intestinal microecology, achieve early prevention and long-term disease control.

## 4 Conclusions and future perspectives

This review mainly found that the genetic variation of host genes has a certain contribution to the composition and abundance of GM. However, in order to quickly adapt to the changing environment, the genome of bacteria is dynamically variable, so that GM has rich intraspecific genetic diversity (Zhernakova et al., 2024). This makes us further consider whether the genetic characteristics of the host will also affect the intraspecific genetic variation of GM, thereby shaping population-specific strains. Therefore, the association between human genetic variation and GM genetic variation still needs more research to explain. In addition, the complexity of GM data makes it difficult for researchers to analyze it, and the statistical test analysis of large quantities of data needs to collect a large number of samples to reduce the error rate. Although GWAS has made great progress in human complex trait genetics and disease microbiology, its method is not fully applicable to the study of host genetics and intestinal microbial interaction (Awany et al., 2019). Therefore, it is still an urgent problem to develop an algorithm to deal with the complex data of microorganisms and to find a new analysis method for GWAS specifically for GM.

Environmental factors are the most easily changed risk factors. Even though it is a great challenge to study the role of environmental factors in the pathogenesis of IBD, it provides special hope for the occurrence and prognosis of IBD. Therefore, deeper research is needed to determine the effects of environmental impacts to determine the role of these factors in causing and mitigating IBD.

Although microbial-derived antigens are essential for activating intestinal immune T cells and B cells, microorganisms and their metabolites also play a key role in guiding the differentiation of these immune cells. Short-chain fatty acids (SCFAs, acetic acid, propionic acid and butyric acid) and bile acids (BAs) are the most common metabolites of GM (Wu J. et al., 2021). Acetic acid is mainly produced by microorganisms such as *Bacteroides* and *Bifidobacterium*. Propionic acid is mainly produced by *Lachnospiraceae* and *Negativicutes*, *Bacteroides* and *Akkermansia*. Butyric acid is the main energy source of colonic epithelial cells, which is produced by *Clostridium bacteria* such as *Faecalibacterium*, *Eubacterium*, *Roseburia* and *Ruminococcus* (Takeuchi et al., 2024). SCFAs affect host immunity through a variety of mechanisms: (1) As a ligand for G protein-coupled receptors (GPRs), SCFAs can regulate the activation and differentiation of immune cells and transmit signals in epithelial cells. (2) SCFAs can regulate gene expression by inhibiting histone deacetylases (HDACs), thereby affecting the function of immune cells. (3) As an energy source, SCFAs can be converted to acetyl-CoA, promoting aerobic respiration and supporting the activation of immune cells (Shan et al., 2022). In addition, the GM affects the composition of BAs and converts primary BAs into secondary BAs through metabolic pathways. These BAs regulate T cell differentiation by interacting with specific receptors, such as inhibiting Th17 and promoting Treg (Takeuchi et al., 2024). Therefore, the next research should focus on a specific intestinal flora and its typical metabolites, and comprehensively elaborate its role in the development of IBD from the aspects of genes, metabolic pathways and metabolites.

In addition, the prevention and treatment of colitis can also be achieved through the GM regulation. Firstly, dynamic tracking of GM changes can provide an understanding of disease progression and is expected to be an effective tool for early screening of colitis. Secondly, in view of the changes of GM composition and function *in vivo*, supplementation with probiotics to alter GM imbalances could provide more personalized and accurate prevention and treatment plans for colitis. Finally, the development of dual-targeted drugs that both modulate specific intestinal microbial abundance and are anti-inflammatory is of great significance for the prevention and treatment of colitis.

## Glossary

***B. fragilis***: *Bacteroides fragilis*
CDI: *Clostridium difficile* infection
DSS: Dextran sodium sulfate
DCs: Dendritic cells
***E. coli***: *Escherichia coli*
***E. faecalis***: *Enterococcus faecalis*
FUT2: Fucosyltransferase 2
***F. nucleatum***: *Fusobacterium nucleatum*
FMT: Fecal microbiota transplantation
GM: Gut microbiota
IBD: Inflammatory bowel disease
***LCT***: Lactase
***MED13L***: Mediator complex subunit 13L
NLRs: NOD-like receptors
NF-κB: Nuclear factor-κB
PRRs: Pattern recognition receptors
SCFAs: Short-chain fatty acids
***S. bovis***: *Streptococcus bovis*
SFB: Segmented filamentous bacteria
TLRs: Toll-like receptors
TLR2mAb: TLR2 monoclonal antibody
TLR4mAb: TLR4 monoclonal antibody
TNF-α: Tumor necrosis factor-α
TGF-β: Transforming growth factor beta
